# Impact of race-based and race-free CKD-EPI equations on estimated glomerular filtration rate in a multicenter retrospective cohort of autosomal dominant polycystic kidney disease

**DOI:** 10.55730/1300-0144.6212

**Published:** 2026-01-25

**Authors:** Özgür Akın OTO, Abdulmecit YILDIZ, İsmail KOÇYİĞİT, Halil YAZICI, Saide Elif GÜLLÜLÜ BOZ, Yiğit KURTULUŞ, Alparslan DEMİRAY, Müge DOKSAN, Vafa SULEYMANOVA, Ece ÜK, Alper AKIN, Bahar BASTANI, John C. EDWARDS, Krista Leigh LENTINE, Yaşar Kerem ÇALIŞKAN

**Affiliations:** 1 Division of Nephrology, Department of Internal Medicine, Faculty of Medicine, İstanbul University, İstanbul, Turkiye; 2 Division of Nephrology, Department of Internal Medicine, Faculty of Medicine, Bursa Uludağ University, Bursa, Turkiye; 3 Division of Nephrology, Department of Internal Medicine, Faculty of Medicine, Erciyes University, Kayseri, Turkiye; 4 Department of Internal Medicine, Faculty of Medicine, İstanbul University, İstanbul, Turkiye; 5 Division of Nephrology, Department of Medicine, Saint Louis University Center for Abdominal Transplantation, Saint Louis, MO, United States

**Keywords:** Autosomal dominant polycystic kidney disease, CKD-EPI equation, estimated glomerular filtration rate, chronic kidney disease staging, race-free equation

## Abstract

**Background/aim:**

The 2021 revision of the Chronic Kidney Disease Epidemiology Collaboration (CKD-EPI) equation, which eliminates race-based coefficients, may significantly influence disease staging and treatment in autosomal dominant polycystic kidney disease (ADPKD). This study was designed to evaluate the impact of the 2021 race-free CKD-EPI equation on estimated glomerular filtration rate (eGFR) and chronic kidney disease (CKD) staging in a multi-ethnic ADPKD population.

**Materials and methods:**

In this multicenter, retrospective cohort study, 597 ADPKD patients were evaluated, including 515 self-identified as Caucasian and 82 as African-American. eGFR values at baseline and final follow-up were calculated using both the 2009 and 2021 CKD-EPI equations. Changes in CKD staging and clinical implications were assessed based on the race-free equation.

**Results:**

Among Caucasian patients, the median baseline eGFR increased from 85.0 to 88.6 mL/min/1.73 m^2^ and the final eGFR from 63.1 to 66.1 mL/min/1.73 m^2^ using the 2021 equation (p < 0.001). In contrast, the 2021 equation resulted in decreased eGFR among African-American patients, from 89.3 to 80.3 mL/min/1.73 m^2^ at baseline and from 46.8 to 42.6 mL/min/1.73 m^2^ at final follow-up (p < 0.001). A total of 6.7% of Caucasian patients were downstaged initially and 5.7% at final follow-up. Conversely, 2.3% of African-American patients were upstaged at baseline and 1.8% at final follow-up.

**Conclusion:**

The 2021 CKD-EPI equation significantly alters eGFR estimation and CKD staging compared to the 2009 version, with varying impacts across ethnic groups. These findings highlight the importance of careful interpretation of eGFR and individualized clinical decision-making in patients with ADPKD.

## 1. Introduction

Glomerular filtration rate (GFR) plays a central role in assessing renal function and in the staging and treatment decision-making process for chronic kidney disease (CKD) [1]. In particular, the Chronic Kidney Disease Epidemiology Collaboration (CKD-EPI) estimated GFR (eGFR) equation is widely used in the management of autosomal dominant polycystic kidney disease (ADPKD); by providing valuable information about kidney function, disease progression, and risk assessment, it guides treatment decisions that impact patient outcomes [2, 3].

The eGFR calculated using a creatinine-alone equation (eGFRcr) from CKD-EPI 2009 and the eGFR calculated using a creatinine and cystatin C equation (eGFRcr-cys) from CKD-EPI 2012 both included ethnic coefficients for both African-American and Caucasian individuals, similar to the modified diet in renal disease (MDRD) and other equations [4]. The newly published 2021 CKD-EPI eGFRcr equation has been redesigned to exclude the ethnicity factor, providing a non-ethnic-based method for eGFR calculation [5]. This 2021 CKD-EPI eGFRcr equation was found to be more accurate than previous equations, with reduced discrepancies between different ethnic groups. However, it has been reported that this new equation without ethnic coefficients slightly underestimates eGFR in African-American people and slightly overestimates eGFR in other cohorts [5,6].

In 2022, U.S. clinical laboratories were advised to promptly adopt the 2021 CKD-EPI eGFRcr equation that was refit without ethnic coefficients [7]. In the European nephrology community, there seems to be a general inclination to continue with the 2009 CKD-EPI equation without applying the African-American race coefficient. This preference is due to observations that the 2021 version tends to overestimate GFR in a significant portion of the European population [8–12]. This stance was influenced by ethical considerations and concerns about the lesser accuracy of GFR estimations made by the 2021 equation.

The 2009 and 2021 CKD-EPI eGFRcr equations have been shown to give different results in the general population [6]. Despite proof of differing outcomes between the 2009 and 2021 CKD-EPI equations in general populations, their comparative effect on specific patient cohorts such as those with ADPKD have not been comprehensively studied. Precise CKD staging in ADPKD is vital for effective disease management, including timing of treatment interventions, patient education, and optimizing clinical trial designs to improve outcomes in this population. Furthermore, CKD staging influences therapeutic decision-making, particularly for newer therapies such as tolvaptan, and guides the timing of renal replacement therapy (RRT) initiation.

This study bridges this gap by assessing the real-world impacts of CKD-EPI 2021 in an international, multiethnic ADPKD cohort, underscoring how the equation changes have impacted CKD staging.

## 2. Material and methods

**Figure 1:**
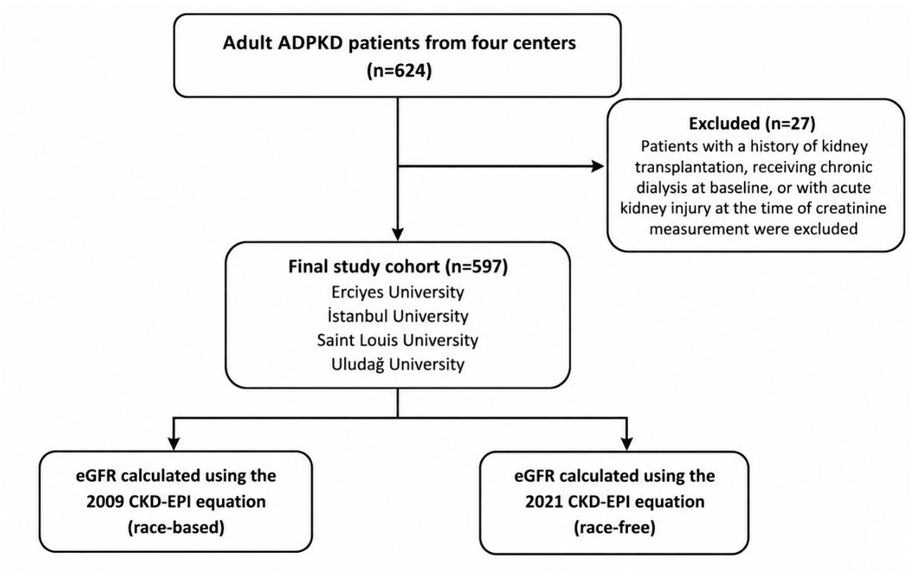
Flow diagram illustrating the study design and patient selection algorithm.

In this multicenter retrospective study, adult patients (aged ≥18 years) diagnosed with ADPKD, as established by accepted radiological criteria and/or genetic testing when available, were evaluated. The study included patients followed at one medical center in the United States and three centers in Türkiye. Demographic, clinical, and laboratory data were retrospectively collected from patient files and electronic hospital records. Eligible patients were required to have available serum creatinine measurements allowing estimation of GFR using both the 2009 and 2021 CKD-EPI equations, with documented baseline and final follow-up values and a minimum follow-up duration of 12 months. A flow diagram summarizing the study design and patient selection algorithm, including inclusion and exclusion criteria, is shown in Figure 1.

Patients with a history of kidney transplantation, those receiving chronic dialysis at baseline, or those with acute kidney injury at the time of creatinine measurement were excluded. To ensure assessment of stable kidney function, serum creatinine values obtained during inpatient care, emergency department visits, or within 24 h of hospital admission were not considered. Patients with incomplete clinical or laboratory data precluding reliable estimation of eGFR, as well as those with concomitant kidney diseases other than ADPKD that could significantly affect renal function, were also excluded from the analysis.

Factors such as hypertension, nephrolithiasis, urinary tract infections, and medication use, including angiotensin-converting enzyme inhibitors (ACEIs), angiotensin receptor blockers (ARBs), and tolvaptan were documented. The eGFR values, calculated using the 2009 [13] and 2021 CKD-EPI [5] equations at the time of initial diagnosis and during the final follow-up were compared to assess their impact on CKD staging [14]. Race and ethnicity were self-ascribed by the patients. Plasma creatinine levels were measured using either enzymatic methods or the corrected Jaffe method.

### 2.1. Statistical analysis

For the statistical analysis, SPSS v.26 (IBM Corp., Armonk, NY) was employed. Continuous variables were tested for normality using the Shapiro–Wilk test. Categorical variables were presented as counts and percentages. T-tests were used to compare groups with parametrically distributed numerical variables and the Kruskal–Wallis test was used for those with non-parametric distributions. For presenting the results, non-parametric variables were summarized using interquartile ranges (IQR 25–75), while parametric variables were expressed as mean ± standard deviation (SD). Due to the retrospective design and heterogeneity in data collection across the participating centers, additional multivariable or sensitivity analyses adjusted for clinical covariates such as age, sex, hypertension, and proteinuria could not be reliably performed. The significance threshold was set at p < 0.05. Ethical approval for this study was obtained from relevant local ethics committees, aligning with the Helsinki Declaration for ethical clinical practices.

## 3. Results

### 3.1. Demographics and clinical parameters

The study comprised 597 ADPKD patients with a median initial age of 37 years (IQR 30.0–46.0) and a slight female predominance (53.1%). Caucasian individuals constituted the majority of the cohort (515, 86.3%), with a median follow-up period of 10 years (IQR 5.0–13.0). Common comorbidities included hypertension (71.0%), nephrolithiasis (27.0%), and urinary tract infection (17.0%). ACEI or ARB were used by nearly half of the patients (49.9%), and a minority were treated with tolvaptan (1.8%), as shown in Table 1. Cerebral aneurysms were recorded only if previously diagnosed or clinically documented, as systematic screening was not performed in the study cohort.

### 3.2. Estimated glomerular filtration rate analysis

A notable divergence in eGFR estimations was observed when comparing the 2009 and 2021 CKD-EPI equations. In the Caucasian patient subgroup, the median initial eGFR increased significantly from 85.0 to 88.6 mL/min/1.73 m^2^ (p < 0.001) using the 2021 CKD-EPI equation. The eGFR obtained during the final follow-up also showed a significant rise, from 63.1 to 66.1 mL/min/1.73 m^2^ (p < 0.001). Conversely, African-American patients had a decrease in the median initial eGFR from 89.3 to 80.3 mL/min/1.73 m^2^ (p < 0.001) and in the final eGFR from 46.8 to 42.6 mL/min/1.73 m^2^ (p < 0.001) with the application of the 2021 CKD-EPI equation (Table 2).

### 3.3. CKD staging transitions among Caucasian patients

**Figure 2:**
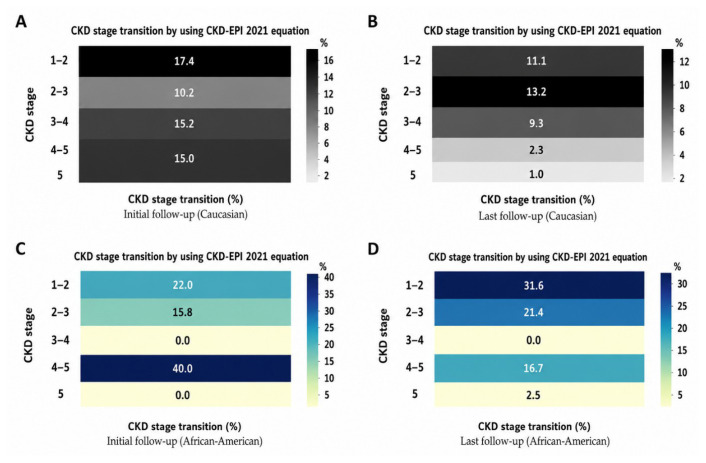
Illustrations of the distribution of CKD stage transitions at initial and final follow-up, stratified by self-identified race. Percentages indicate the proportion of patients transitioning between CKD stages over time. (A) CKD stage transition percentages at initial follow-up in Caucasian patients. (B) CKD stage transition percentages at final follow-up in Caucasian patients. (C) CKD stage transition percentages at initial follow-up in African-American patients. (D) CKD stage transition percentages at final follow-up in African-American patients. CKD = chronic kidney disease and CKD-EPI = chronic kidney disease epidemiology collaboration.

The comparative analysis of CKD staging transitions using the 2021 CKD-EPI equation for Caucasian patients with ADPKD indicated a general trend towards a reduction in disease severity. Initially, 40 Caucasian patients (6.7%) were downstaged, and at the final follow-up, 34 patients (5.7%) were at a lower stage when reassessed with the 2021 CKD-EPI equation. In the initial assessment using the 2009 CKD-EPI equation, 21 (17.4%) in stage 2, 11 (10.2%) in stage 3, five (15.2%) in stage 4, and three (15.0 %) in stage 5 were assigned to a lower stage (Figure 2A). In the final staging comparison, 12 (11.1%) in Stage 2, 15 (13.2%) in Stage 3, four (9.3%) in Stage 4, and three (3.4%) in Stage 5 were assigned to a lower stage with the 2021 CKD-EPI equation (Figure 2B).

### 3.4. CKD staging transitions among African-American patients

In contrast to the Caucasian patients, the African-American ADPKD patients tended to be reclassified to a more advanced stage of CKD with the 2021 equation. Initially, 14 African-American patients (2.3%) were upstaged, and at the final evaluation, 11 patients (1.8%) were at a higher stage. Using the 2009 CKD-EPI equation, initially nine (22.0%) in stage 1, three (15.8%) in stage 2, none in stage 3, and two (40.0%) in stage 4 were transitioned to a higher stage according to the 2021 equation (Figure 2C). In the final CKD staging, six (31.6%) in stage 1, three (21.4%) in stage 2, none in stage 3, and two (16.7%) in stage 4 were reclassified to a more advanced stage with the 2021 CKD-EPI equation (Figure 2D).

## 4. Discussion

This study’s assessment of the 2009 and 2021 CKD-EPI equations in Caucasian and African-American ADPKD populations revealed significant changes in eGFR estimations and consequent CKD staging. The Caucasian subgroup experienced an increase in median eGFR when transitioning from the 2009 CKD-EPI to the 2021 equation, indicating a trend towards milder disease classification. Initial and final eGFR readings showed statistically significant rises, with several patients being downstaged in their disease severity according to the new equation.

Conversely, the African-American cohort displayed a decrease in median eGFR values with the 2021 equation, suggesting an escalation in disease severity. This was reflected in a notable percentage of patients being reclassified to more advanced stages of CKD in both the initial and final assessments.

In a population-based study conducted in Sweden, the effects of using the 2021 CKD-EPI creatinine equation in Europe’s predominantly Caucasian population were examined. The study found that switching to the 2021 equation led to an average increase in eGFR of 3.9 mL/min/1.73 m^2^, significantly impacting older individuals and men. This transition resulted in the reclassification of 9.9% of the total population and 36.2% of CKD G3a-G5 patients to a higher eGFR category. Despite these changes, eGFR remained a strong predictor of outcomes, with both the 2021 and 2009 equations demonstrating similar accuracy and calibration in estimating kidney failure risk [15].

In a relatively recent study from China, the eGFR was calculated using the 2009 and 2021 CKD-EPI equations based on the initial creatinine levels of 1,051,827 participants [4]. The findings indicated that the use of the 2021 equation generally increased eGFR by 4.46% compared to the 2009 equation, with 85.89% of participants having a higher eGFR, yet this change did not lead to a modification in CKD stage. Conversely, while 11.57% of the group showed an improvement in CKD stage, only 0.75% had lower eGFR values, but this also did not result in a change in CKD stage. These results demonstrate that the 2021 CKD-EPI equation generally yields higher eGFR results compared to the 2009 version, and it can lead to changes in CKD stage in some cases.

The recalibration of eGFR from the 2009 CKD-EPI to the 2021 CKD-EPI equation has engendered substantive changes in the staging of CKD, particularly in the context of ADPKD. This reevaluation of renal function holds profound implications for therapeutic decision-making and the overall clinical trajectory for patients.

Particularly in the African-American cohort, some patients initially classified as stage 4 CKD under the 2009 CKD-EPI equation might be reclassified to stage 5 with the 2021 CKD-EPI equation.

Reclassification of CKD stages following the application of the 2021 CKD-EPI equation may have important implications for disease-modifying therapy in ADPKD, particularly regarding eligibility for tolvaptan treatment. As treatment initiation and continuation are closely linked to eGFR thresholds, downward or upward shifts in CKD stage driven solely by equation choice may influence clinical decision-making independent of true changes in kidney function. This is especially relevant for patients near treatment cutoffs, where reclassification could lead to delayed initiation or premature discontinuation of therapy.

Changes in eGFR may also affect decisions regarding the use of contrast-enhanced imaging modalities, such as contrast-enhanced computed tomography or magnetic resonance imaging, which are frequently required for clinical evaluation and management of ADPKD. Equation-driven reductions in eGFR could increase caution or avoidance of contrast use, potentially limiting diagnostic or interventional options, whereas upward reclassification may result in greater perceived safety despite unchanged underlying renal function.

Finally, CKD stage reclassification may influence the timing of RRT planning and access to kidney transplantation. Patients reclassified to more advanced CKD stages may undergo earlier preparation for dialysis or be prioritized for transplant evaluation, while those shifted to less advanced stages may experience delays in referral or access to transplant waiting lists. These findings highlight that changes in CKD staging associated with different eGFR equations may have downstream clinical consequences that extend beyond risk stratification and should therefore be interpreted cautiously in the management of ADPKD.

This study is the first to compare the 2009 and 2021 CKD-EPI equations within a specific population with ADPKD, providing critical insights into how these updated equations impact disease staging in this specialized group and enhancing our understanding of ADPKD management.

Previous studies have clearly demonstrated the limitations of eGFR equations in ADPKD. Ruggenenti et al. reported that 2009 CKD-EPI creatinine-based equation significantly overestimated GFR when compared with iohexol-measured GFR and failed to reliably detect longitudinal changes in kidney function, with prediction errors often exceeding the magnitude of true GFR decline [16]. Similarly, a recent comprehensive evaluation comparing 61 creatinine- and cystatin C–based equations showed that none achieved acceptable agreement with measured GFR across the spectrum of kidney function in ADPKD, with frequent misclassification around clinically relevant thresholds for treatment eligibility and reimbursement [17]. In this context, the higher eGFR values observed with the 2021 CKD-EPI equation in Caucasian patients in this study’s cohort should not be interpreted as reflecting improved accuracy or true biological preservation of kidney function. Rather, these findings underscore equation-dependent variability and the inherent unreliability of creatinine-based estimates in ADPKD. These results therefore emphasize that changes in CKD staging following adoption of the 2021 CKD-EPI equation primarily reflect methodological differences between equations and may amplify over- or underestimation of renal function, with potentially important clinical consequences when fixed eGFR thresholds guide therapeutic decisions.

One significant limitation of this study is the lack of directly measured GFR (mGFR) using the gold standard reference method. Therefore, these findings do not allow the drawing of definitive conclusions about the accuracy or superiority of the 2021 CKD-EPI equation compared to the 2009 CKD-EPI equation in ADPKD. This study was designed to evaluate the clinical and staging effects of applying different eGFR equations to the same serum creatinine values in a real-world ADPKD cohort. Thus, the observed differences in estimated GFR and CKD stage reclassification should be interpreted as equation-induced effects rather than actual functional changes in renal function.

In addition to its retrospective design, this study does not address the effects of reclassification on medication choice and the timing of RRT initiation. Another important limitation of this study is the relatively small number of African-American patients included in the cohort. Furthermore, racial groups other than Caucasian and African-American were underrepresented in the study population. This may limit the generalizability of subgroup analyses and CKD stage transition findings, so these results should be interpreted with caution. The serum creatinine measurements were performed using different assay methods (enzymatic or Jaffe) across centers, which may have introduced variability in the eGFR calculations and should be considered as a methodological limitation. Although initial and final eGFR values were available, assessment of eGFR slope over follow-up could not be reliably performed due to the retrospective design and lack of standardized intermediate measurements across centers, which is an additional limitation of the study. Another limitation of this study is the potential for center-related and population-based bias, as patient distribution across centers may have differed by ethnicity, with African-American patients predominantly recruited from the United States and Caucasian patients from Türkiye. Finally, the study did not include the European Kidney Function Consortium or other emerging equations.

In conclusion, the application of the 2021 CKD-EPI equation in ADPKD patients does not reflect a uniform improvement or deterioration in kidney function. Rather, it results in differential changes in estimated GFR across ethnic groups, leading to clinically relevant shifts in CKD staging. These equation-driven differences may influence therapeutic eligibility, clinical decision-making, and timing of RRT initiation, underscoring the need for cautious interpretation of eGFR values in the management of ADPKD.

## Figures and Tables

**Table 1:** Demographic, clinical, and laboratory parameters of patients with autosomal dominant polycystic kidney disease.

Parameter	Value
Initial age, years, median, (IQR 25–75)	37.0 (30.0–46.0)
Final age, years, median, (IQR 25–75)	47.0 (40.0–54.0)
Sex, male/female, n (%)	280/317 (46.9/53.1)
Race, Caucasian/African-American	515/82 (86.3/13.7)
Follow-up period, years, median, (IQR 25–75)	10.0 (5.0–13.0)
Family history, n (%)	369 (61.9)
Smoking, n (%)	219 (36.7)
BMI, kg/m^2^, median, (IQR 25–75)	26.0 (24.0–30.0)
Nephrolithiasis, n (%)	129 (27.0)
Macroscopic hematuria, n (%)	72 (15.1)
Cerebral aneurysm, n (%)	9 (1.9)
Urinary infection, n (%)	81 (17.0)
Hepatic cyst, n (%)	172 (39.7)
Hypertension, n (%)	424 (71.0)
ACEI/ARB use, n (%)	238 (49.9)
Tolvaptan use, n (%)	11 (1.8)
Initial serum creatinine, mg/dL, median (IQR 25–75)	0.9 (0.7–1.2)
Initial proteinuria, mg/g, median (IQR 25–75)	120.0 (80.0–200.0)
Final serum creatinine, mg/dL, median (IQR 25–75)	1.2 (0.9–1.8)
Final proteinuria, mg/g, median (IQR 25–75)	160.0 (91.0–180.0)

IQR = interquartile range, BMI = body mass index, ACEI= angiotensin-converting enzyme inhibitors, and ARB = angiotensin II receptor blockers.

**Table 2:** Comparative analysis of GFR estimates using CKD-EPI 2009 and CKD-EPI 2021 equations in Caucasian and African-American populations.

		CKD-EPI 2009	CKD-EPI 2021	p-value
Caucasian				
	Initial eGFR (mL/min/1.73 m^2^)	85.0 (52.0–107.8)	88.6 (55.1–111.3)	<0.001
	Final eGFR (mL/min/1.73 m^2^)	63.1 (29.1–98.2)	66.1 (30.6–102.5)	<0.001
African-American				
	Initial eGFR (mL/min/1.73 m^2^)	89.3 (54.6–120.8)	80.3 (50.3–106.9)	<0.001
	Final GFR (mL/min/1.73 m^2^)	46.8 (12.8–85.3)	42.6 (11.9–76.4)	<0.001

eGFR = estimated glomerular filtration rate and CKD-EPI = chronic kidney disease epidemiology collaboration.
